# On‐line untargeted metabolomics monitoring of an *Escherichia coli* succinate fermentation process

**DOI:** 10.1002/bit.28173

**Published:** 2022-07-15

**Authors:** Joan Cortada‐Garcia, Jennifer Haggarty, Tessa Moses, Rónán Daly, Susan Alison Arnold, Karl Burgess

**Affiliations:** ^1^ Institute of Quantitative Biology, Biochemistry and Biotechnology, School of Biological Sciences University of Edinburgh Edinburgh UK; ^2^ Institute of Infection, Immunity and Inflammation, Glasgow Polyomics University of Glasgow Glasgow UK; ^3^ EdinOmics, SynthSys—Centre for Synthetic and Systems Biology, School of Biological Sciences The University of Edinburgh Edinburgh UK; ^4^ Ingenza Ltd., Roslin Innovation Centre Roslin UK

**Keywords:** bioprocess monitoring, fermentation monitoring, on‐line metabolomics, real‐time metabolomics, succinate

## Abstract

The real‐time monitoring of metabolites (RTMet) is instrumental for the industrial production of biobased fermentation products. This study shows the first application of untargeted on‐line metabolomics for the monitoring of undiluted fermentation broth samples taken automatically from a 5 L bioreactor every 5 min via flow injection mass spectrometry. The travel time from the bioreactor to the mass spectrometer was 30 s. Using mass spectrometry allows, on the one hand, the direct monitoring of targeted key process compounds of interest and, on the other hand, provides information on hundreds of additional untargeted compounds without requiring previous calibration data. In this study, this technology was applied in an *Escherichia coli* succinate fermentation process and 886 different *m*/*z* signals were monitored, including key process compounds (glucose, succinate, and pyruvate), potential biomarkers of biomass formation such as (*R*)‐2,3‐dihydroxy‐isovalerate and (*R*)‐2,3‐dihydroxy‐3‐methylpentanoate and compounds from the pentose phosphate pathway and nucleotide metabolism, among others. The main advantage of the RTMet technology is that it allows the monitoring of hundreds of signals without the requirement of developing partial least squares regression models, making it a perfect tool for bioprocess monitoring and for testing many different strains and process conditions for bioprocess development.

## Graphical abstract

Untargeted metabolomics was used in this study to monitor an *Escherichia coli* fermentation process of succinate production, achieving the automatic analysis of 886*m*/*z* signals every 5 min from a bench‐top 5 L bioreactor. Cortada‐Garcia and co‐workers describe how this technology can be used to find key bioprocess compounds and monitoring biomarkers, as well as help identify relevant pathways for the bioprocess without the requirement of developing partial least squares regression models, making it a perfect tool for bioprocess development.

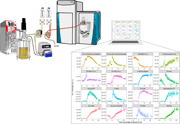

## 1 INTRODUCTION

### 1.1 Fermentation monitoring

Fermentation monitoring is a pivotal tool for bioprocess optimization and control. Assessing the state of the process via high‐resolution time‐course analysis allows detailed characterization during process development, as well as rapid detection and correction of any possible deviations from desired process specifications during product manufacturing, ensuring the quality of the end product (Svendsen et al., 2015; Zu et al., 2017).

Different parameters are frequently monitored in fermentation processes, such as the pH, temperature, and concentration of dissolved oxygen (DO) in the liquid phase, as well as the oxygen and carbon dioxide in the gas phase. Although there are many other parameters that can be measured—such as turbidity, rheology, enzyme activity, or metabolite concentration among others (Harada et al., 2014)—their monitoring is less common, especially at a large scale.

In a bioreactor, the biomass and bioprocess metabolites—such as substrates and products—are found in the liquid phase. The monitoring of these compounds has received a lot of attention in the last decades. Different technologies can be used to monitor the biomass, including optical density (turbidity), dielectric spectroscopy, microscopy, flow cytometry, fluorescence spectroscopy, calorimetry, and vibrational spectroscopy (Bayer et al., 2020; Broger et al., 2011; Grobbelaar, 2009; Kamiloglu et al., 2020; Müller et al., 2018; Sonnleitner, 2012), whereas the monitoring of metabolites can be achieved using high‐performance liquid chromatography (HPLC), vibrational spectroscopy, nuclear magnetic resonance (NMR), enzymatic reactions, and mass spectrometry (MS) (Druhmann et al., 2011; Shalabaeva et al., 2017; Svendsen et al., 2015; Vann et al., 2017; Warth et al., 2010). As metabolites are at the final step of biological regulation, monitoring them provides the best picture of cellular phenotypes (Farrell et al., 2014; Fiehn, 2002). Metabolite and transcriptional changes can occur very rapidly. For instance, Xu et al. (2012) detected changes in glycolysis and tricarboxylic acid (TCA) cycle metabolites within 1–5 min of removing or changing the carbon source in the growth medium, and Lara et al. (2006) calculated the time required to synthesize one molecule of messenger RNA of the mixed‐acid fermentation genes to be between 10 and 72 s. For this reason, monitoring the metabolites in a bioprocess via high‐resolution time‐course analysis enables the detection of these fast metabolic changes much earlier than using conventional off‐line analysis, which is usually sparse, in the order of magnitude of hours.

Bioreactor monitoring by HPLC has been implemented on‐line, but it has some limitations such as time delays between samples of typically around 10 min and the requirement of a biomass filtration system to avoid column blocking (Koch et al., 2016; Koliander et al., 1990; Warth et al., 2010), limiting the analysis to extracellular metabolites.

Vibrational spectroscopy—especially near‐infrared, mid‐infrared, and Raman spectroscopy—has been implemented in‐line and on‐line, yielding very accurate monitoring models for several process compounds. However, these vibrational spectroscopy technologies have certain limitations, the main one being that the spectra that they generate are very convoluted with many overlapping signals. This results in the need to use chemometric mathematical models such as partial least squares (PLS) regression to break down the different signals contributed by the different compounds in the mixture (do Nascimento et al., 2017; Li et al., 2018; Marison et al., 2012; Rodrigues et al., 2018; Stuart, 2005; Zu et al., 2017). These models require significant time and resources to build and are usually not transferable, that is, they are only applicable to the configuration used to build them (bioreactor, medium composition, strain, temperature, pH, etc.) (Marison et al., 2012; Pu et al., 2020; Roggo et al., 2007), making these monitoring techniques of limited use for early stages of bioprocess development, when the strain, process parameters, and media composition are often changed in an iterative manner (Baradez et al., 2018). Finally, due to the large signal overlap, these technologies tend to report only a few compounds from the mixture, usually the most abundant ones. There have been some examples in the literature using NMR for on‐line fermentation monitoring (Kreyenschulte et al., 2015; Legner et al., 2019). Similar to vibrational spectroscopy, a limitation of NMR for bioprocess monitoring is the presence of overlapping peaks, which limits the number of compounds that can be detected and quantified, usually less than 10 (Brecker et al., 1999; Kreyenschulte et al., 2015; Majors et al., 2008).

Due to the increasing demand for tools to monitor metabolites, a range of commercial bioprocess analyzers has been developed in the last couple of decades. Some of these are based on enzymatic analysis—such as the Cedex Bio® Analyzer (Roche) and the BioProfile FLEX2 (Nova biomedical) (Morris et al., 2021; Obaidi et al., 2021)—while others use the so‐called “miniaturized” MS analyzers—such as the MiD (Microsaic) and the Rebel (908 devices) (Hamilton et al., 2014; Synoground et al., 2021), employing a low‐resolution quadrupole and ion trap mass analyzers, respectively (Blakeman & Miller, 2021; Hemida et al., 2021). However, these analyzers are still almost exclusively being used at‐line or off‐line—thus limiting their monitoring potential—and only targeting the predefined set of compounds dictated by the vendor reagents.

The aim of this study is to explore the use of untargeted metabolomics as a technology to monitor the metabolites present in the liquid phase of a bioreactor in real time. Metabolomics is the global analysis of small to medium size molecules (i.e., up to 1000–2000 Da) present in the metabolism of biological systems. Current analytical methods and MS instrumentation allow for a generous compound coverage across different metabolic pathways, thus facilitating the interpretation of biological experiments. The advantages of MS are that it offers a much wider detection capacity than NMR, vibrational spectroscopy, the common detectors used with HPLC (refractive index [RI] and UV/Vis spectroscopy) and commercial enzymatic analyzers, has very high sensitivity and allows detection of metabolites in a much less convoluted manner than NMR and vibrational spectroscopy, thus not requiring the development of laborious chemometric models such as PLS regression. All these attributes make MS an attractive technology for bioprocess monitoring.

Despite these advantages, to date, on‐line metabolomics still remains unexploited for bioprocess monitoring. Link et al. (2015) reported the use of metabolomics to monitor different organisms directly from a cultivation flask. However, the cells grown in these experiments were cultured in fermentation media that was up to eight times diluted, which is an impractical imitation for bioprocess monitoring. Plum and Rehorek (2005) reported an on‐line MS system for analyzing nine azo dyes in a wastewater treatment process. However, this system contained a biomass filtration unit and targeted only nine compounds, thus limiting the vast detection capacity of MS.

**Figure 1:**
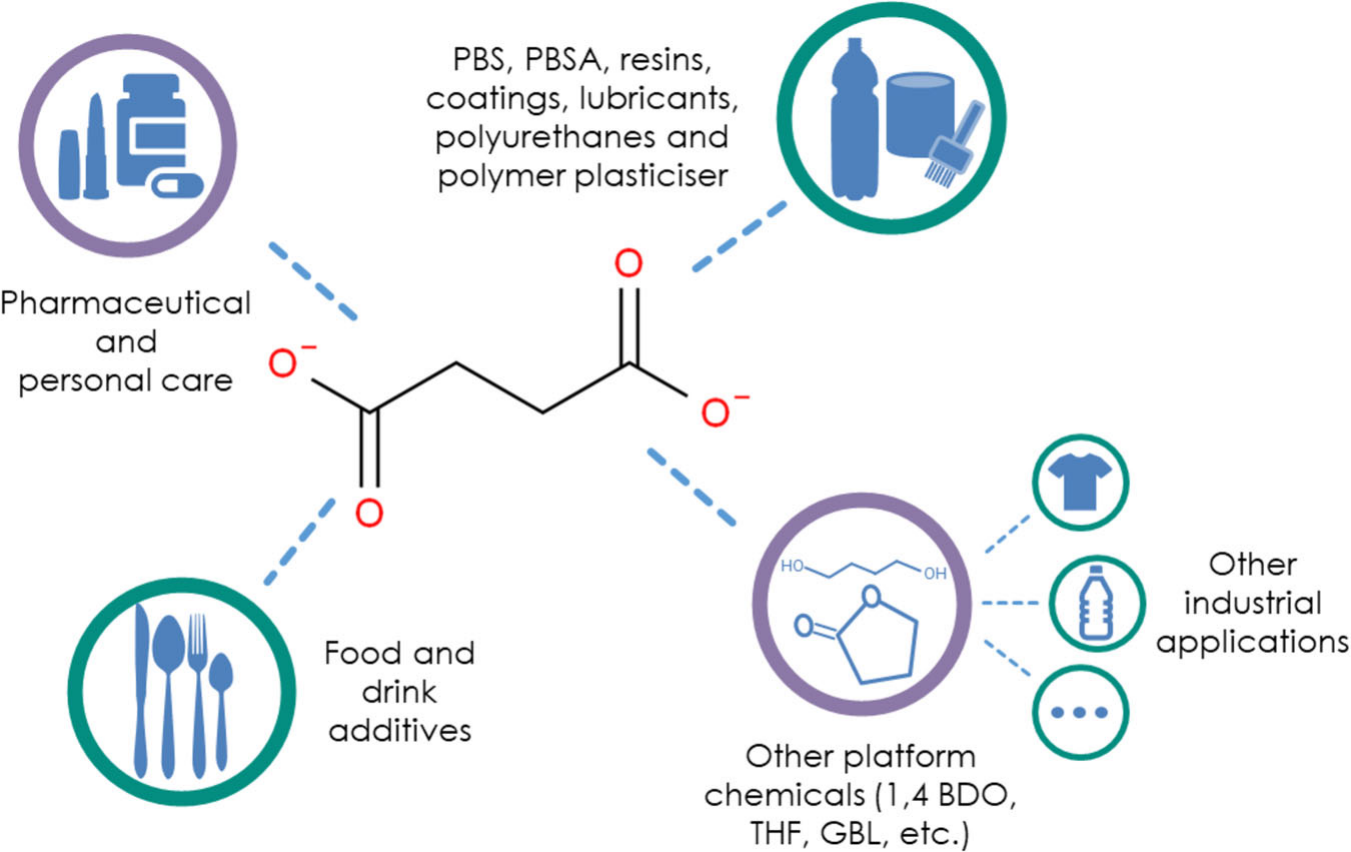
Industrial applications of succinic acid. 1,4‐BDO, 1,4‐butanediol; GBL, γ‐butyrolactone; PBS, polybutylene succinate; PBSA, polybutylene succinate adipate; THF, tetrahydrofuran.

In this study, untargeted metabolomics has been used for the first time, to our knowledge, for bioprocess monitoring. This technology was tested with a bench‐top 5 L bioreactor using undiluted and unfiltered fermentation medium for the untargeted monitoring of 886 different intracellular and extracellular *m*/*z* signals of an *Escherichia coli* (*E. coli*) succinate fermentation process using a high‐resolution Orbitrap mass spectrometer. Succinate is used as an intermediate in the manufacturing of high‐value consumer products such as personal care items, pharmaceutical intermediates and food and drink additives, as well as in the manufacturing of high production‐volume products such as polybutylene succinate (PBS), polybutylene succinate adipate (PBSA), resins, coatings, lubricants, and polyurethanes. Furthermore, succinate can also be derivatised into other platform chemicals such as 1,4‐butanediol, tetrahydrofuran, and γ‐butyrolactone (Matano et al., 2014; Nghiem et al., 2017; Saxena et al., 2017; Thakker et al., 2012) (see Figure 1), all of which have significant market applications, such as the production of elastic fibers, plastics, and polyurethanes. Detected features include, among others, the main process compounds, potential biomarkers of biomass formation and metabolites from the pentose phosphate pathway (PPP) and nucleotide metabolism. This study is a step in the development of new technology for both bioprocess monitoring during product manufacturing, and also for earlier research and development phases; for instance, for the evaluation of different strains, process conditions and for the identification of engineering targets, by‐products, and biomarkers, among others.

## 2 MATERIALS AND METHODS

### 2.1 Bacterial strain

All experiments described in this article were carried out using a proprietary industrial *E. coli* strain (Ingenza Ltd.), based on the *E. coli* NZN111 strain with deletions of the pyruvate‐formate lyase (*pflB*) and lactate dehydrogenase (*ldhA*) genes as described by Chatterjee et al. (2001).

### 2.2 Growth media

All 5 L scale fermentation experiments were carried out with a batch phase for biomass formation using a defined minimal medium containing 11.90 g/L glucose as the sole carbon source, 2.00 mM MgSO_4_, a mix of salts solution (2.00 g/L (NH_4_)_2_SO_4_, 14.60 g/L K_2_HPO_4_, 3.60 g/L NaH_2_PO_4_·2H_2_O, 0.50 g/L (NH_4_)_2_H‐citrate), a mix of trace elements (1.0 mg/L CaCl_2_·2H_2_O, 20.06 mg/L FeCl_3_, 0.36 mg/L ZnSO_4_·7H_2_O, 0.32 mg/L CuSO_4_·5H_2_O, 0.30 mg/L MnSO_4_·H_2_O, 0.36 mg/L CoCl_2_·6H_2_O, 44.60 mg/L Na_2_EDTA·2H_2_O), antibiotics (100 mg/L kanamycin, 34 mg/L chloramphenicol), and antifoam (33.33 µl/L polypropylene glycol P‐2000). Shake flask overnight cultures were prepared using the same medium but with 10.00 g/L glucose and no antifoam.

### 2.3 Fermentation process conditions

**Figure 2:**
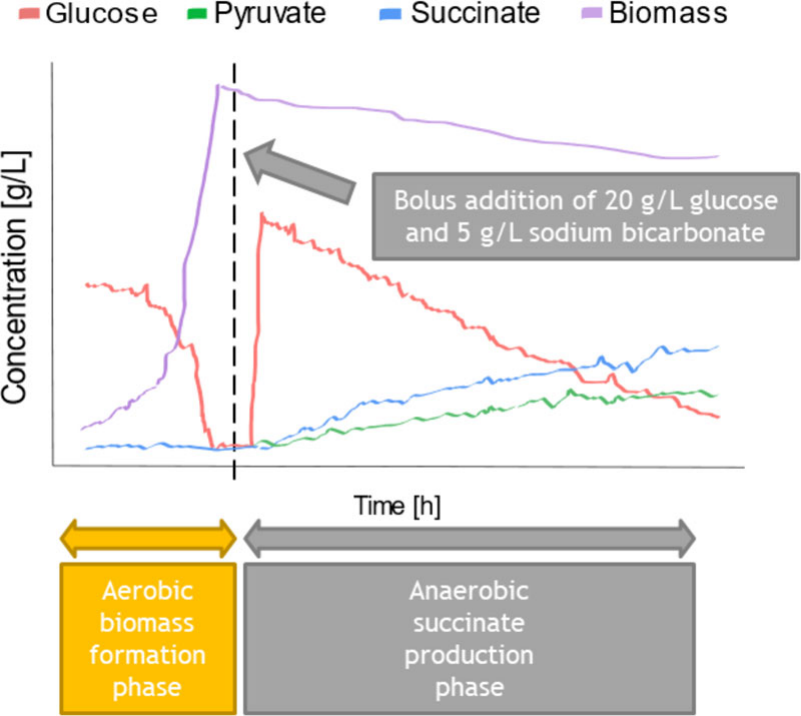
Schematic diagram of the fermentation process. The dashed black line splits both phases of the process.

All fermentation experiments were carried out in a 5 L Applikon stirred tank fermenter (ADI 1030 Bio Controller, 1035 Bio Console), and the process consisted of an initial batch phase where the minimal medium was primarily used for biomass formation, followed by a 24 h anaerobic succinate production phase (Figure 2), similar to the process described by Vemuri et al. (2002).

#### 2.3.1 Inoculum

Fermentation inocula were prepared by inoculating 50 µl of cell bank into 100 ml of growth medium in a 500 ml baffled shake flask and incubated at 37°C and 165 rpm for 17–17.5 h.

#### 2.3.2 Aerobic batch phase for biomass growth

The fermentation was started by inoculating 100 ml of overnight culture into 3 L of growth medium in the 5 L fermenter for a starting OD_600_ of 0.21 ± 0.025. During biomass growth, the conditions were maintained at 37°C temperature, 500–900 rpm agitation (controlled to keep the DO > 30%), 4.00 L/min air (1.33 vvm), and pH 7.0 ± 0.1, controlled with 2.00 M H_2_SO_4_ and 28% (w/v) NH_4_OH.

#### 2.3.3 Anaerobic succinate production phase

**Figure 3:**
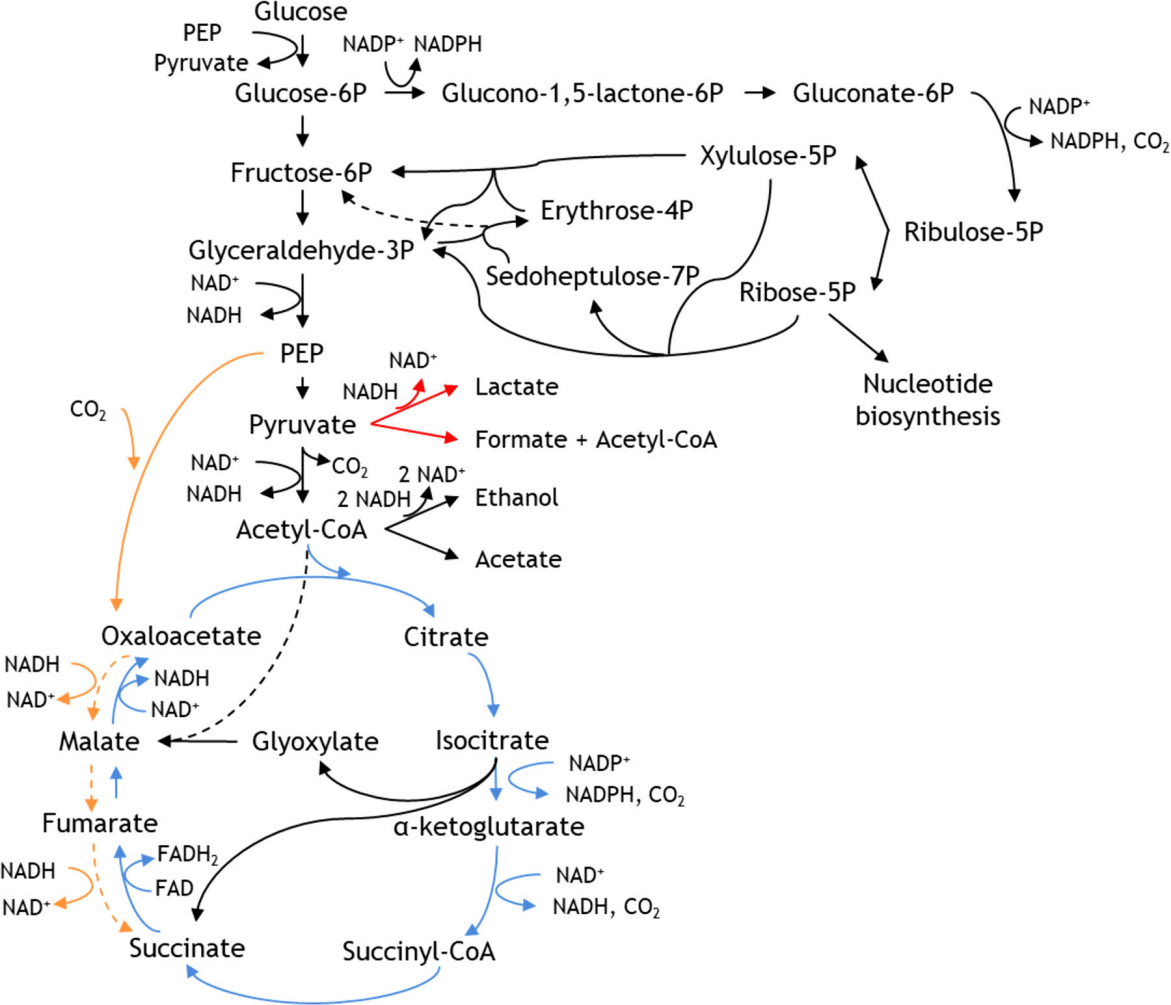
Main metabolic reactions involved in succinate production in Escherichia coli. Blue lines indicate the oxidative TCA cycle under aerobic conditions. Orange lines indicate the reductive TCA cycle under anaerobic conditions. Red lines indicate deleted reactions in the industrial strain used. Arrows crossing other reactions are marked with black dashes

At the beginning of the production phase, glucose from a 500 g/L solution and sodium bicarbonate from a 100 g/L solution were added to the fermenter as a single bolus addition to a final concentration of 20 and 5 g/L, respectively, in the vessel, as described by Wu et al. (2007). The sodium bicarbonate provides soluble CO_2_, which is required for the conversion of PEP to oxaloacetate (Figure 3) (Thakker et al., 2012). Once the glucose and sodium bicarbonate were added to the fermenter, the sparged air was replaced by pure (99.8%) CO_2_ at 0.50 L/min (0.17 vvm), agitation was set to 300 rpm, temperature at 37°C, and pH at 7.0 ± 0.1, controlled with 2.00 M H_2_SO_4_ and 28% (w/v) NH_4_OH.

### 2.4 Biomass measurement

Biomass levels were reported as OD_600_ and wet cell weight (WCW). The former was the measured optical density at 600 nm wavelength. The latter was determined by spinning down 1 ml of sample for 5 min at 14,462*g* twice in a preweighed Eppendorf tube, removing the supernatant and weighing the resulting pellet. The weight of the pellet in g/L was calculated from gravimetric difference.

### 2.5 On‐line metabolomics

**Figure 4:**
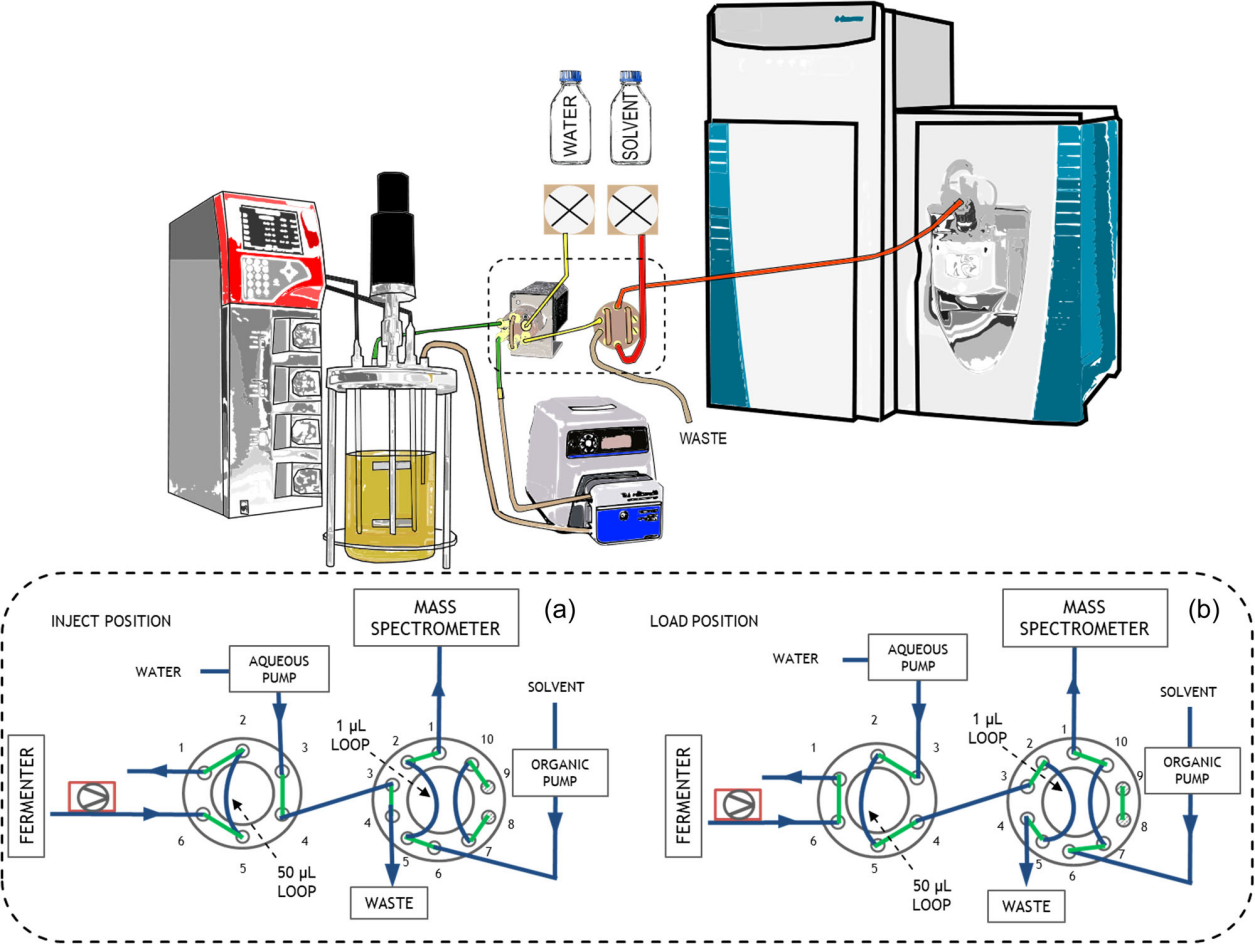
Experimental setup of the on‐line metabolomics system (top) and a detailed diagram of the dashed area showing the two valve positions (bottom). During the “inject position”, fermentation broth sample is continuously circulated through the 50 µl of the left six‐port valve (sample *n* + 1) and the fermentation broth sample from the 1 µl loop in the right 10‐port valve (sample *n*) is pushed to the mass spectrometer. During the “load position,” the sample *n* + 1 is pushed from the 50 µl loop of the six‐port valve into the 1 µl loop of the 10‐port valve, ready for injection at the next “Inject position.”

On‐line metabolomics was conducted by connecting the fermenter to an Exactive™ Orbitrap (Thermo Scientific) mass spectrometer with a fluidics system similar to what had previously been described in the literature (Link et al., 2015), but adapted to inject undiluted fermentation broth samples straight into the mass spectrometer. The modified fluidics system consisted of a peristaltic pump and two valves (Figure 4), and sample injections were alternated with washing steps, one‐to‐one. The peristaltic pump was a Masterflex™ L/S® (Cole‐Parmer) high‐performance pump model 77252‐72 and was operated at a high flow rate of 75–100 ml/min. The first valve was a six‐port, two‐position valve (Vici Valco®) and the second one was a 10‐port, two‐position valve (Dionex Corporation). Note, however, that no chromatography was used.

#### 2.5.1 Sample injections

During sample injections, fermentation broth containing cells is constantly extracted from the fermenter with the peristaltic pump, injected into the six‐port valve, circulated through a 50 µl loop and returned to the fermenter. Upon valve switching, the broth sample from the 50 µl loop of the six‐port valve is carried by sterile water pumped at a 200 µl/min flow rate using an external piston pump into the 10‐port valve, where it is collected in a 1 µl loop. The sample is finally injected into the mass spectrometer carried by a 70:30 ACN:IPA + 0.1% formic acid mixture at a 400 µl/min flow rate. The duration of the injection method was 1 min, and the total traveling time from the bioreactor to the mass spectrometer was 30 s, with ca. 10 s to reach the six‐port valve and 20 more seconds to reach the mass spectrometer.

#### 2.5.2 Washing steps

Each sample injection was followed by a 4 min washing step to avoid system blockage and signal loss. The six‐port valve was washed with 70:30 IPA:ACN + 0.1% formic acid for 1 min and with sterile water for 3 min, both at a 400 µl/min flow rate. The 10‐port valve was washed with sterile water for 2 min and then 70:30 ACN:IPA + 0.1% formic acid for 2 min, both at a 600 µl/min flow rate (see Supporting Information: Figure S1). The washing solutions were sent to waste and did not enter either the fermenter or the mass spectrometer.

#### 2.5.3 Mass spectrometer parameters

Gas‐phase ions were generated with an electrospray ionization (ESI) source. The mass spectrometer was operated at 50,000 resolution, mass range 50–1000*m*/*z* in polarity switching mode with a spray voltage of ±3.5 kV. The capillary temperature was set to 350°C, sheath gas 40 a.u., automatic gain control target 1 × 10^6^ a.u., and the lock masses in the positive and negative mode were 74.0964*m*/*z* and 112.9856*m*/*z*, respectively.

### 2.6 Metabolomics data processing and analysis

Raw MS data were processed with the Xcalibur™ software (version 3.1.66.10) using the Genesis peak detection method. The peak integration threshold was set to 0.5 signal‐to‐noise ratio (S/N), smoothing points to 1 and peak detection was set to the highest peak within a 15 s retention window, with a minimum peak height threshold of 3 S/N. A small number of signals that were not properly detected with the Genesis method were instead processed with the ICIS method. In these cases, peak integration was performed setting smoothing points to 1, baseline window to 40, area noise factor to 5, peak noise factor to 10, minimum peak height threshold of 3 S/N and peak width constrained to 5% of the peak height with a tailing factor of 2.

After processing the raw data with the Xcalibur™ software, metabolite features were extracted as a.csv file, which was used to generate time‐course metabolic profiles using the ggplot2 package (version 3.3.3; Wickham, 2016) in the statistical software environment R (version 3.6.1). Data smoothing was carried out using the same version of the ggplot2 package with a locally estimated scatterplot smoothing method with a span between 0.2 and 0.5, depending on the metabolite.

### 2.7 HPLC‐UV/Vis‐RI analysis

HPLC coupled to UV/Vis and RI detectors (HPLC‐UV/Vis‐RI) analysis was carried out using a Rezex™ ROA Organic Acid H + ion‐exclusion column (Phenomenex®) (300 mm × 7.8 mm) equipped with a Carbo‐H4 guard column (SecurityGuard™) (3.0 mm i.d.). An isocratic method was applied to the column, running a 5 mM H_2_SO_4_ mobile phase solution for 30 min. The total flow rate was 800 µl/min, column temperature was maintained at 65°C, sample injection volume was 10 µl, and samples were maintained at 4°C for the duration of the analysis. The HPLC‐UV/Vis‐RI data were extracted as a.csv file and were further analyzed using the ggplot2 package (version 3.3.3; Wickham, 2016) in the statistical software environment R (version 3.6.1).

### 2.8 Correlation analysis between off‐line and real‐time metabolomics data

A Pearson test was used to analyze the correlation between offline data (HPLC‐UV/Vis‐RI and WCW) and real‐time metabolomics data. This test was performed using the ggpubr package (version 0.4.0; Kassambara, 2020) in the statistical software environment R (version 4.0.4).

### 2.9 Data scaling

Data scaling was done by applying Equation (1), where “y” is any given time‐course vector, such as the MS intensity of a metabolite throughout the duration of the fermentation. After scaling, the scaled data will range from 0 to 1,

(1) $$y_{\mathrm{norm}}=\frac{y-\min(y)}{\max\left(y\right)-\min(y)}.$$

## 3 RESULTS AND DISCUSSION

### 3.1 Development of an on‐line MS system

An on‐line MS system was developed consisting of the following key components: a peristaltic pump, a six‐port valve, and a 10‐port valve, as graphically depicted in Figure 4.

#### 3.1.1 Peristaltic pump

Fermentation broth containing cells and medium is continuously pumped out of the bioreactor through a peristaltic pump at a high flow rate (75–100 ml/min) to avoid the accumulation of biomass in the system and to minimize the traveling time of the sample from the fermenter into the mass spectrometer. It was observed that a high‐pressure peristaltic pump was necessary to prevent the malfunctioning of the pump mid‐way through a fermentation experiment. The monitoring system handles whole‐broth samples containing cells. The back pressure of the pumps increases during the sampling and decreases during the washing step. Due to the increase in pressure, it is better to have a pump that can withstand a higher pressure. A Masterflex™ L/S® (Cole‐Parmer) high‐performance pump model 77252‐72 was chosen for this purpose.

#### 3.1.2 Six‐port valve

The fermentation broth is pushed by the peristaltic pump into a six‐port valve, which has a 50 µl sampling loop. The broth is recirculated back into the fermenter for the majority of the time in the “inject position” (see Figure 4). When the valve position is changed to “load position,” the 50 µl of the sampling loop is pushed with sterile water at 200 µl/min using an external pump and travels to the 10‐port valve, where it is collected in a 1 µl loop. A final valve switch to the “inject position” injects the sample into the mass spectrometer carried by a 70:30 ACN:IPA + 0.1% formic acid solvent mixture at a 400 µl/min flow rate using a second external pump. A VICI® Cheminert® 6 port two‐position valve is used in the on‐line system. A wide‐bore diameter valve (0.75 mm) was chosen to minimize the risk of blockages from the biomass.

#### 3.1.3 10‐Port valve

During the “load position,” the 50 µl fermentation broth sample collected in the sampling loop of the six‐port valve is introduced into the 10‐port valve, pushed with sterile water using a piston pump from an HPLC instrument (aqueous pump). This way, the fermentation sample is delivered to a 1 µl sampling loop on the 10‐port valve. When the valve switches to the “inject position,” the 1 µl sample gets injected into the mass spectrometer, pushed with a 70:30 ACN:IPA + 0.1% formic acid solvent mixture using a second piston pump from the same HPLC instrument (organic pump).

#### 3.1.4 Further considerations of the system

Using two valves is a solution to mitigate the solvent incompatibility at the two ends of the system. Namely, at one end, the bioreactor contains a water‐based environment with living cells, and at the other end, ESI MS works best with volatile organic solvents, which are more effective than water at generating gas‐phase ions (Hoffmann & Stroobant, 2007).

The total traveling time from the bioreactor to the mass spectrometer was 30 s, with ca. 10 s to reach the six‐port valve and 20 more seconds to reach the mass spectrometer. This traveling time is short compared to the biomass doubling time (65 min, see Supporting Information: Figure S2) and allows the capture of rapid metabolic changes while minimizing the time the sample spends out of the fermentation environment.

#### 3.1.5 Introduction of a washing step between sample injections

A washing step between sample injections was introduced to prevent blockages of the on‐line monitoring system. By tracking the total ion chromatogram (TIC) across the first injections of two different fermentation experiments, it was observed that the washing step was instrumental in preventing signal loss of the mass spectrometer. Specifically, it was observed that when one wash was performed after every three samples, the TIC signal consistently increased immediately after every washing step (Supporting Information: Figure S3A). This indicated that the washing step helps to prevent not only system blockages but also signal loss, potentially due to the removal of particulates and build‐up molecules accumulated in the system during sample injection. When the washing step was used after every injection (Supporting Information: Figure S3B), the changes in the TIC did not follow any periodic pattern. In both cases (with a wash after three samples and a wash after each sample), there was a decreasing trend in the TIC during the first 15 injections, but this is probably not caused by signal loss, but by the consumption of glucose from the media by the cells for biomass formation. With these observations, it was deemed necessary to implement a washing step after every single injection.

### 3.2 On‐line untargeted metabolomics analysis of a succinate fermentation process

**Figure 5:**
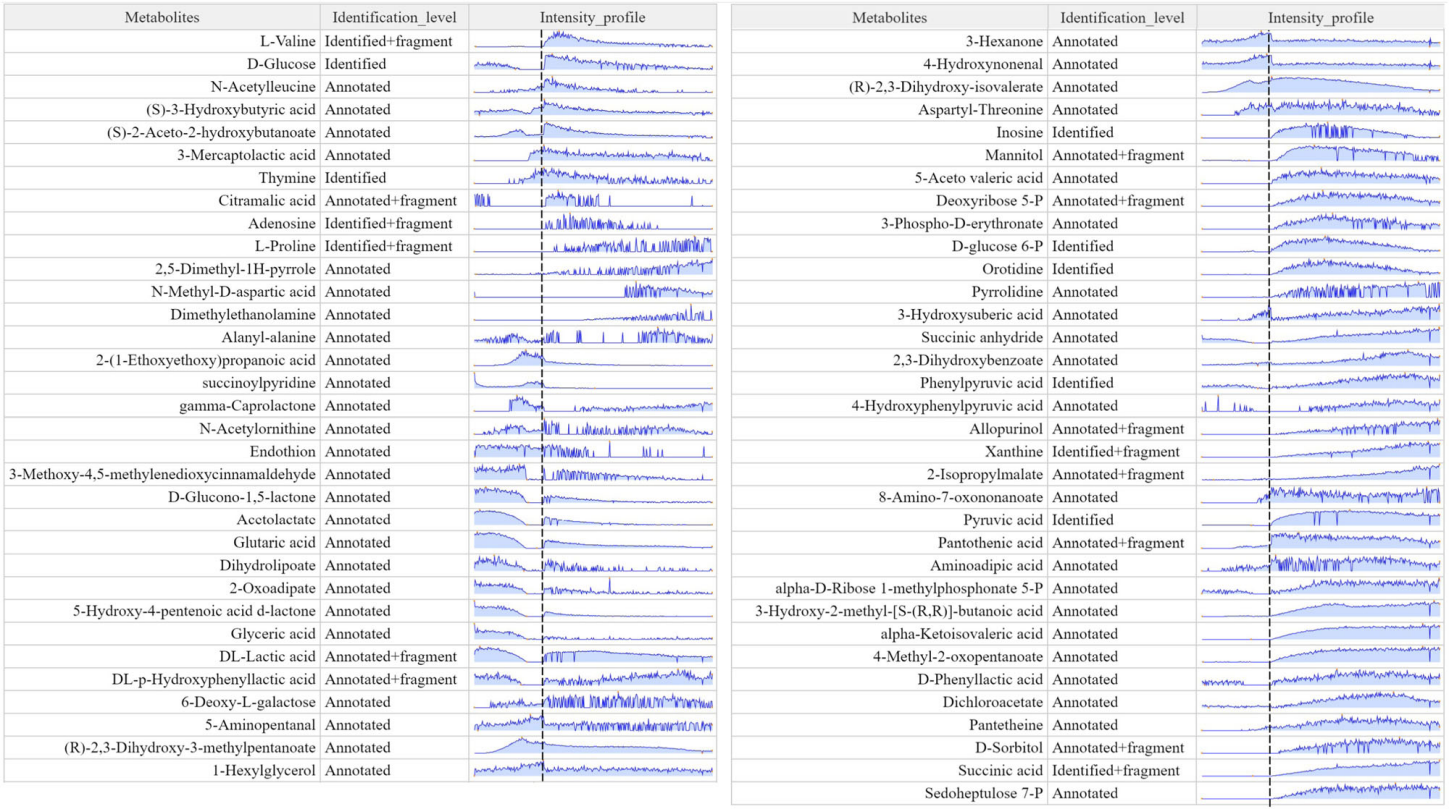
Signals monitored with on‐line metabolomics matching the time evolution profile and accurate mass of the results obtained by liquid chromatography‐mass spectrometry analysis with 5 ppm error. For easier visualization, the signals were scaled and then clustered according to their patterns. The vertical black dashed line indicates the transition from the aerobic growth phase to the anaerobic succinate production phase. Noisy signals are caused by low ion intensity values, close to the detection threshold (1000 a.u.).

**Figure 6:**
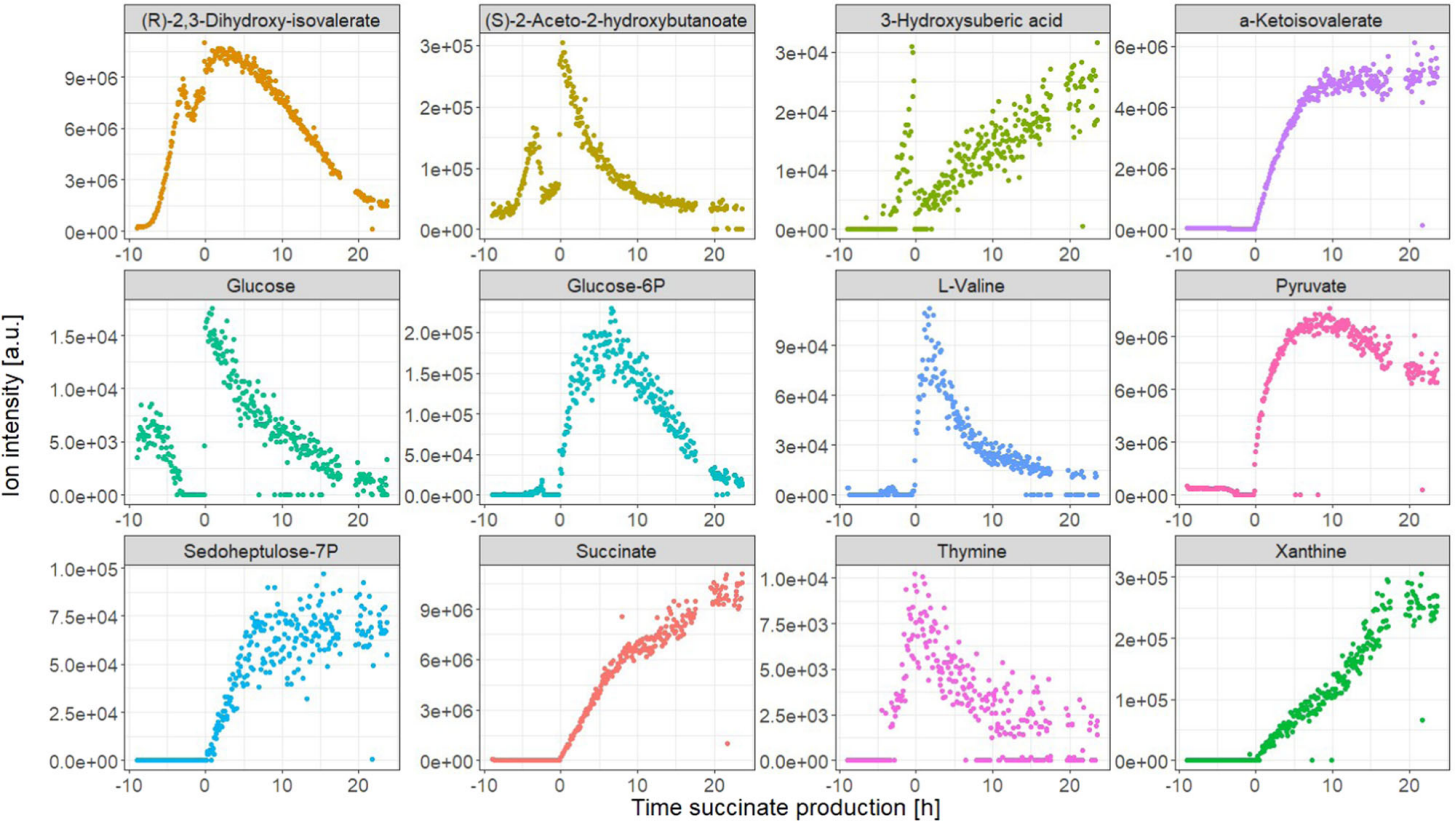
Example of annotated metabolites observed with on‐line metabolomics monitoring of a succinate production fermentation process in *Escherichia coli*. Time is indicated with respect to the beginning of the succinate production phase.

Nine fermentation runs were performed testing different parameters of the on‐line monitoring system—the solvent system, the washing method, sampling frequency, and wash frequency (see Supporting Information: Table S1). The best conditions were selected based on being able to run the fermentation process without blockage or overpressure of the system. The monitoring system operated under the best conditions tested could monitor a total of 359 and 527 different features in negative and positive mode, respectively (886 in total) of the *E. coli* succinate fermentation process. From these, 124 signals were annotated as different metabolites based on accurate mass within 5 ppm *m*/*z* error, and 67 of these were matched to annotations performed by off‐line liquid chromatography‐MS (LC‐MS) analysis (Figure 5) (see Supporting Information: Material for more details about the LC‐MS method). Figure 6 shows some indicative profiles described by different metabolites. The data show some metabolites such as (*R*)‐2,3‐dihydroxy‐isovalerate and (*S*)‐2‐aceto‐2‐hydroxybutanoate to follow an exponential increase in intensity during the biomass formation phase, indicating that these could be used as biomass biomarkers for on‐line monitoring. Another observation is that sugar phosphates involved in the PPP, such as glucose‐6P and sedoheptulose‐7P, and some compounds involved in nucleotide metabolism, such as thymine and xanthine, show a significant increase in intensity at the beginning of the succinate production phase. These patterns can help identify relevant pathways for the bioprocess and can help direct metabolic engineering strategies for process optimization.

The on‐line data also offers a very high time‐resolution compared to the full fermentation duration, allowing identification of key points of the bioprocess with a 5‐minute error margin, such as glucose depletion (2.95 h before succinate production) and the beginning of succinate production.

### 3.3 On‐line metabolomics compared to off‐line HPLC‐UV/Vis‐RI analysis

**Figure 7:**
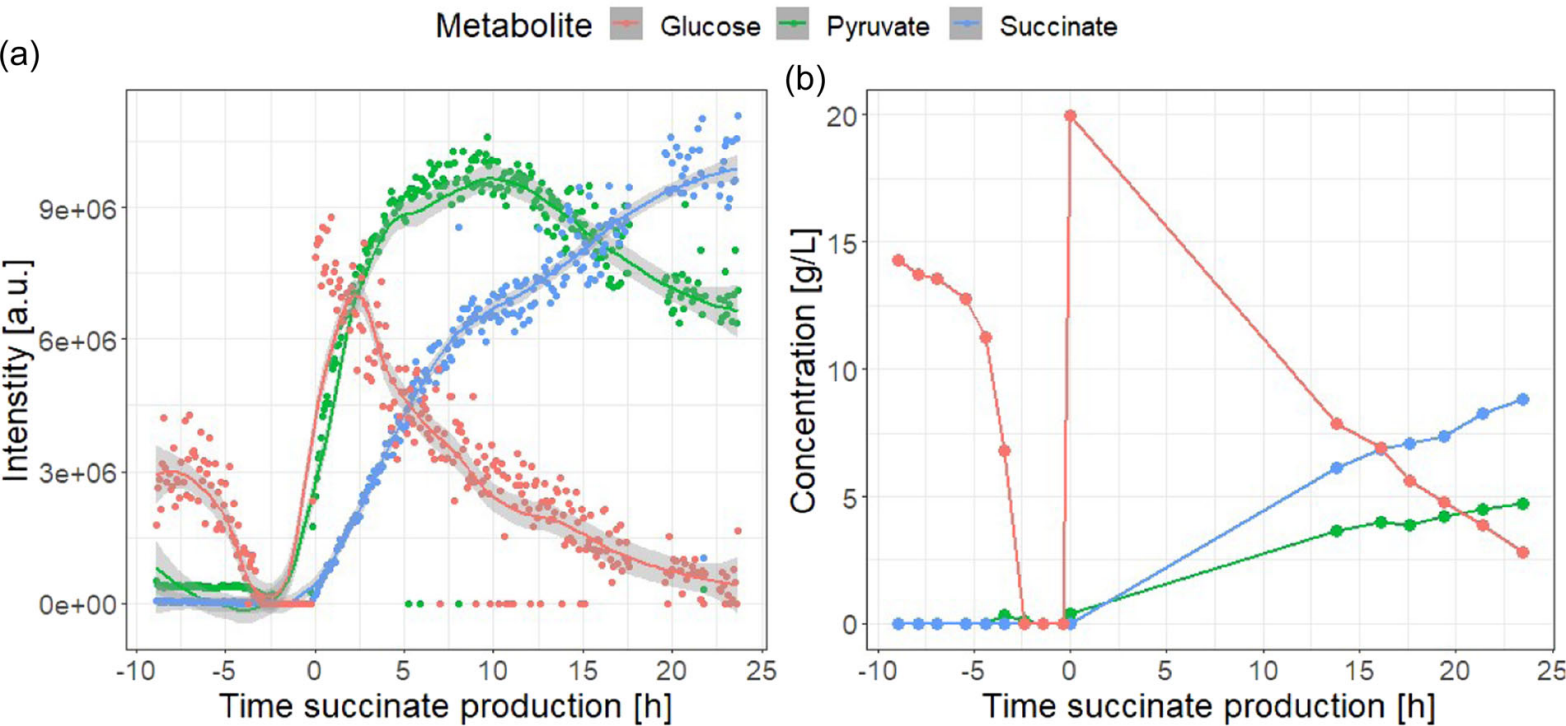
Glucose, pyruvate, and succinate are monitored by on‐line metabolomics (a) and off‐line high‐performance liquid chromatography (HPLC) (b). The mass spectrometry measurements are represented as dots and the corresponding smoothed signal is represented with lines and calculated with locally estimated scatterplot smoothing. Ions 203.0527, 87.0088, and 117.0193*m*/*z* were, respectively, used for glucose, pyruvate, and succinate. The HPLC data are represented as dots and the interpolated data are represented with lines. Time is indicated with respect to the beginning of the succinate production phase.

HPLC is the most widely used reference method for fermentation off‐line analysis and it is commonly used for validation of monitoring methods (Cabaneros Lopez et al., 2019; Legner et al., 2019; Rodrigues et al., 2018). Off‐line samples from the fermentation experiment from Figure 6 were analyzed by HPLC coupled to UV/Vis and refractive index detectors (HPLC‐UV/Vis‐RI) and compared to the on‐line metabolomics signals for the key process metabolites present in both data sets—glucose, succinate, and pyruvate (Figure 7). Comparing the off‐line HPLC‐UV/Vis‐RI results with the on‐line metabolomics data demonstrates the main advantages of real‐time metabolomics. On the one hand, the off‐line data consists of 16 time points, taken on an hourly basis and with time gaps of more than 8 h corresponding to overnight periods, whereas with on‐line metabolomics, samples were automatically collected every 5 min, resulting in 355 time points (a 22‐fold increase in resolution as compared to off‐line analysis). Furthermore, HPLC‐UV/Vis‐RI is run as a targeted analysis, leading to the analysis of only a handful of compounds, whereas, as demonstrated in Figures 5 and 6, metabolomics can be used to monitor a much wider range of metabolites.

**Figure 8:**
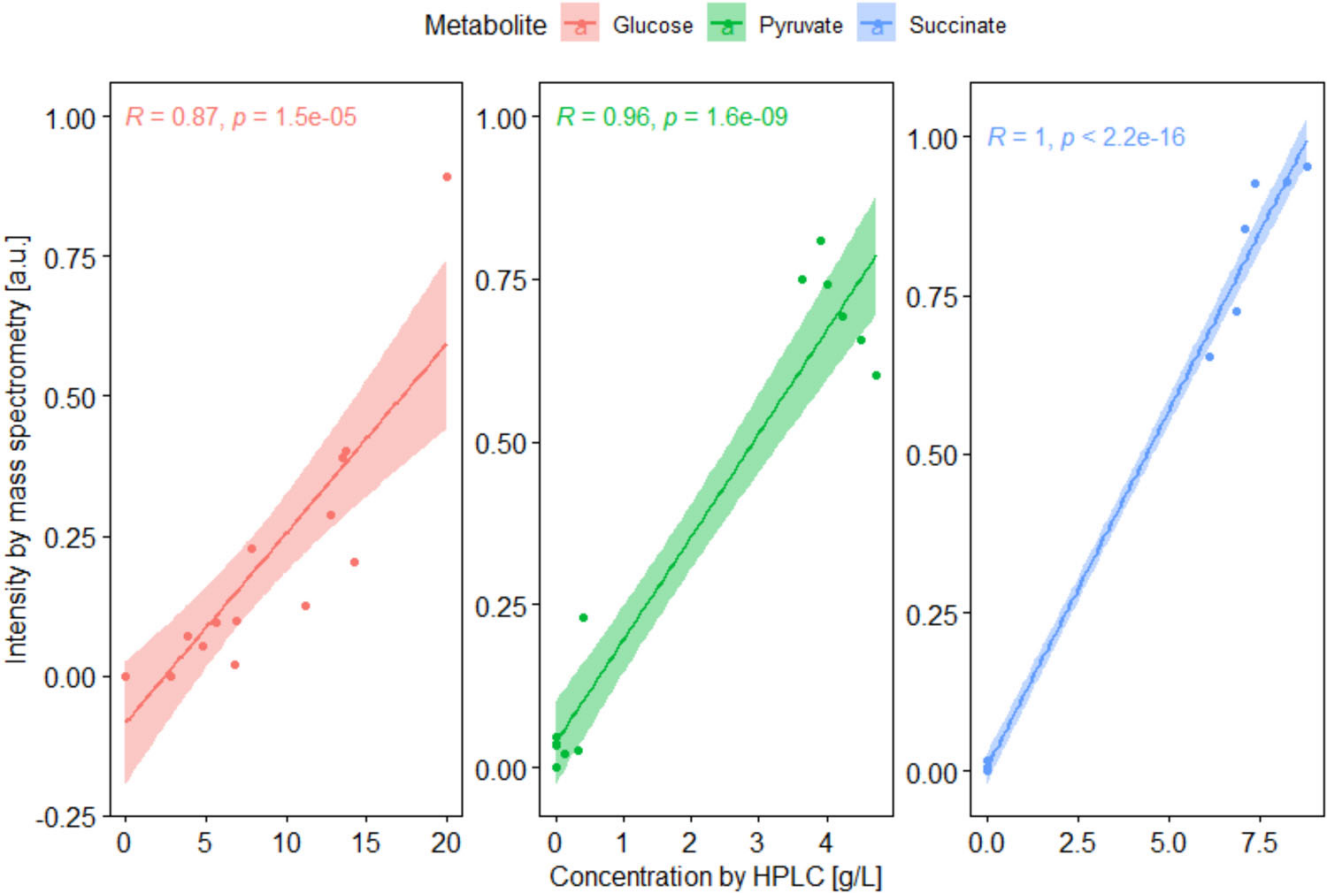
Pearson correlation between off‐line HPLC‐UV/Vis‐RI (refractive index) and on‐line metabolomics data for glucose, pyruvate, and succinate, where R is the Pearson correlation coefficient and *p* shows the *p* value of the test. The metabolomics data were scaled from 0 to 1 to fit in the same axis.

Figure 8 shows that the Pearson correlation estimates between the two data sets were 0.86, 1.00, and 0.96 for glucose, succinate, and pyruvate, respectively, showing a good positive correlation between the data collected with both methods. This study demonstrates how compounds of interest for the bioprocess can be identified in an untargeted manner with the wide detection capacity of metabolomics, while also providing the opportunity for the discovery of unexpected key metabolites.

### 3.4 Biomarkers for growing biomass

**Figure 9:**
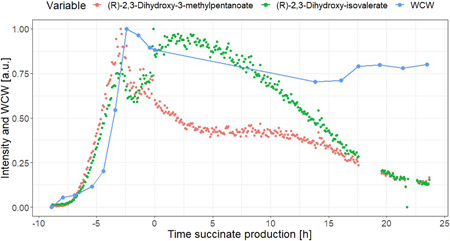
On‐line metabolomics signals corresponding (*R*)‐2,3‐dihydroxy‐3‐methylpentanoate and (*R*)‐2,3‐dihydroxy‐isovalerate and of‐line WCW biomass, all three scaled from 0 to 1 to fit in the same axis. Time is indicated with respect to the beginning of the succinate production phase.

**Figure 10:**
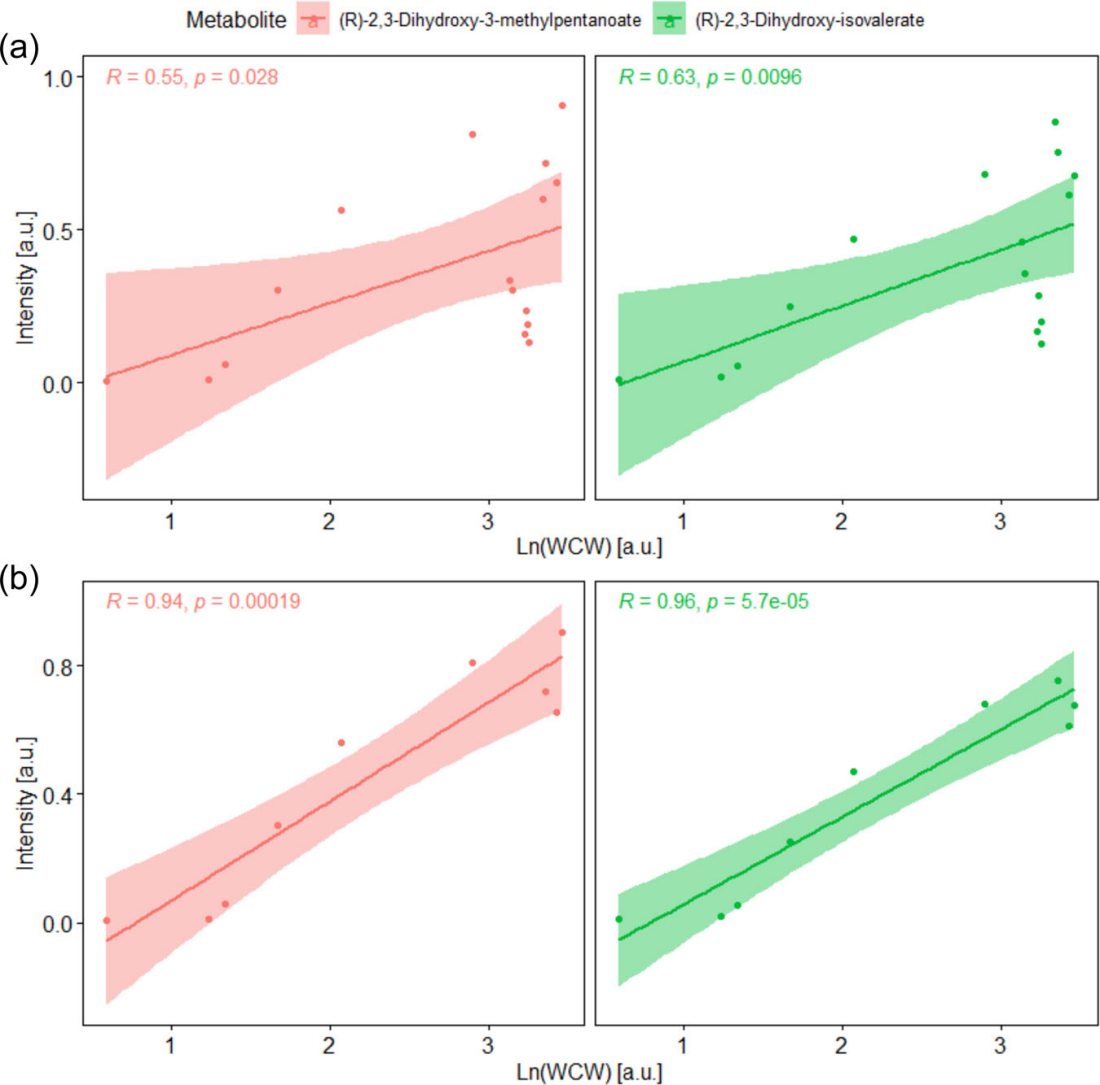
Pearson correlation between the natural logarithm of the WCW biomass and real‐time metabolomics data for (*R*)‐2,3‐dihydroxy‐3‐methylpentanoate and (*R*)‐2,3‐dihydroxy‐isovalerate using the data for the whole fermentation (a) or only using the data of the aerobic batch phase of biomass growth (b). *R* is the Pearson correlation coefficient and p shows the *p* value of the test.

Metabolites annotated as (*R*)‐2,3‐dihydroxy‐3‐methylpentanoate and (*R*)‐2,3‐dihydroxy‐isovalerate follow an exponential increase coinciding with the exponential growth of biomass during the batch phase (Figure 9). For this reason, these two metabolites were identified as potential biomarkers for biomass. To evaluate this, the on‐line signal of these two metabolites was compared with the off‐line WCW biomass measurements by Pearson correlation (Figure 10). A better correlation was found using the natural logarithm of the biomass WCW. When the whole fermentation was evaluated, the correlation between the two signals was poor (Pearson correlation estimates 0.55 and 0.63; Figure 10a). However, a good correlation was found during the aerobic batch phase (Pearson correlation estimates 0.94 and 0.96; Figure 10b), suggesting that (*R*)‐2,3‐dihydroxy‐3‐methylpentanoate and (R)‐2,3‐dihydroxy‐isovalerate, especially the latter, could potentially be used as biomarkers for growing biomass. Both these metabolites belong to the branched‐chain amino acid biosynthetic pathways for the formation of valine, leucine and isoleucine—essential building blocks for biomass formation—which could explain the good correlation of these metabolites with biomass growth.

## 4 CONCLUSIONS

Fermentation monitoring is a crucial step to understand and control the evolution of a bioprocess to ensure that the desired process specifications are met during product manufacturing. For this reason, the monitoring of metabolites in the liquid phase of bioreactors has received increasing attention in the biotech industry in the last couple of decades. MS presents several advantages compared to other technologies that have been more extensively reviewed in the literature, such as on‐line HPLC and vibrational spectroscopy. Namely, MS can detect many more compounds, has a higher sensitivity, and does not require the use of PLS regression models, which tend to have little transferability when process conditions are changed (e.g., temperature, medium, strain, etc.).

Commercial enzymatic analyzers and “miniaturized” low‐resolution mass spectrometers are also becoming a trend for bioprocess analysis, allowing the rapid measurement of a predefined set of compounds. However, these are still almost exclusively used at‐line or off‐line, requiring manual handling and offering limited time resolution. Furthermore, these systems are limited to the analysis of the compounds in the commercial assay kits, and the use of low‐resolution MS significantly limits the quality of metabolite annotation.

In this study, an on‐line untargeted metabolomics platform (RTMet) was developed to be able to analyze fermentation whole‐broth samples directly from the bioreactor with flow injection MS (no chromatography) every 5 min and no manual intervention. The use of a high‐resolution Orbitrap mass spectrometer allowed for the detection of 67 compounds without the need to build time‐consuming PLS regression models. These features make this technology especially useful for the detection of important pathways, by‐products, and biomarkers during the process development stage, allowing the evaluation of different strains, cell lines, and process conditions (temperature, medium, pH, etc.). This is the first step in demonstrating the use of untargeted on‐line metabolomics for bioprocess optimization. Future work will include the use of this technology with other bioprocesses and organisms, as well as the development of quantitative monitoring models to be able to correlate ion intensity to metabolite concentration.

## AUTHOR CONTRIBUTIONS

Karl Burgess conceived the idea and obtained the funding. Joan Cortada‐Garcia and Jennifer Haggarty did the experimental work and analyzed the data. Karl Burgess, Joan Cortada‐Garcia, and Jennifer Haggarty made substantial contributions to the development of the on‐line metabolomics system. Joan Cortada‐Garcia wrote the manuscript and drew the figures. Susan Alison Arnold, Karl Burgess, Rónán Daly, and Tessa Moses provided critical revision of the manuscript and made substantial contributions to its writing. Susan Alison Arnold, Karl Burgess, and Rónán Daly supervised the project and provided intellectual contributions.

## Supporting information

Supplementary information.

## Data Availability

The data that support the findings of this study will be openly available in MetaboLights at https://www.ebi.ac.uk/metabolights/index, reference number MTBLS5197.
