# Supplementary material for: On‐line untargeted metabolomics monitoring of an Escherichia coli succinate fermentation process
Source: Biotechnol Bioeng. 2022 Jul 15;119(10):2757–69. doi: 10.1002/bit.28173 (PMC9541951; doi:10.1002/bit.28173)
Supplement: Supplementary file 1 — Supplementary information. [file BIT-119-2757-s001.docx]

Supporting Information

On-line untargeted metabolomics monitoring of an E. coli succinate fermentation process

Joan Cortada-Garcia ^1^, Jennifer Haggarty ^2^, Tessa Moses ^3^, Rónán Daly ^2^, S. Alison Arnold ^4^ and Karl Burgess ^1^

1 Institute of Quantitative Biology, Biochemistry and Biotechnology, School of Biological Sciences, University of Edinburgh, Edinburgh EH8 9AB, United Kingdom
2 Institute of Infection, Immunity and Inflammation, Glasgow Polyomics, University of Glasgow, Glasgow G61 1QH, United Kingdom
3 EdinOmics, SynthSys - Centre for Synthetic and Systems Biology, School of Biological Sciences, The University of Edinburgh, Edinburgh EH9 3BF, UK
4 Ingenza Ltd., Roslin Innovation Centre, Roslin EH25 9RG, United Kingdom

Table of contents

[SUPPLEMENTARY MATERIALS AND METHODS S1](#_Toc99118423)

[Extractions for off-line LC-MS analysis S1](#_Toc99118424)

[Off-line LC-MS analysis S2](#_Toc99118425)

[Off-line LC-MS data processing and analysis S3](#_Toc99118426)

[SUPPLEMENTARY FIGURES S4](#_Toc99118427)

[SUPPLEMENTARY TABLES S6](#_Toc99118428)

SUPPLEMENTARY MATERIALS AND METHODS

Extractions for off-line LC-MS analysis

Samples for off-line liquid chromatography-mass spectrometry (LC-MS) analysis were spun down twice at 4 °C and 13,000 g for 10 min immediately after being removed from the bioreactor. The supernatant and cell pellet were collected as extracellular and intracellular fractions respectively and stored at -80 °C until further extraction for LC-MS analysis.

Extracellular fractions

Extracellular fraction extractions were prepared by diluting approximately 10 µL of sample into 400 µL of 1:3:1 chloroform:methanol:water (C:M:W). The samples were then mixed vigorously in a chilled microtube mixer for 5 min and then centrifuged for 3 min at 13,000 g and 4 °C. At this point, 360 µL of supernatant were transferred into a new microtube and stored at -80 °C until LC-MS analysis. 25 µL of supernatant of each extracted sample were combined into one single vial to generate a pooled sample. During handling, the 1:3:1 C:M:W extraction solvent and the samples were kept on an ethanol dry ice bath.

Intracellular fractions

Prior to extraction, intracellular fractions were washed by resuspending the cell pellets in 1 mL of sterile phosphate buffer solution. The phosphate buffer was removed by spinning down the samples twice for 10 min at 13,000 g and 4 °C. For metabolite extraction, 200 µL of 1:3:1 C:M:W were added for every 5 mg of WCW pellet. Cell pellets were resuspended by pipetting, and then the samples were mixed vigorously in a chilled microtube mixer for 1 h, before being centrifuged for 3 min at 13,000 g and 4 °C. At this point, 200 µL of supernatant were transferred into a new microtube. The samples were further diluted by adding 200 µL 1:3:1 C:M:W and stored at -80 °C until LC-MS analysis. 25 µL of supernatant of each extracted sample were combined into one single vial to generate a pooled sample. During handling, the 1:3:1 C:M:W extraction solvent and the samples were kept on an ethanol dry ice bath.

Off-line LC-MS analysis

Metabolite separation was performed using a zwitterionic hydrophilic interaction liquid chromatography (ZIC®-pHILIC) column (Merck SeQuant®) (150 mm x 4.6 mm, 5 µm particle size) equipped with the corresponding guard column (20 mm x 2.1 mm, 5 µm particle size) (Merck SeQuant®). A linear gradient was applied to the column, running from 80 to 20 % solvent B over 15 min, followed by a 2 min wash with 5 % solvent B, and 9 min re-equilibration with 80 % solvent B, where solvent B was acetonitrile and solvent A (the remaining percentage) was 20 mM ammonium carbonate in water. The total flow rate was 300 µL/min, column temperature was maintained at 25 °C, sample injection volume was 10 µL, samples were maintained at 4 °C for the duration of the analysis and a HESI probe was used on the ion source.

Metabolite detection was done in a high-resolution Thermo Scientific^TM^ Q Exactive^TM^ Orbitrap mass spectrometer at 70,000 resolution, mass range 70 – 1,050 m/z in polarity switching mode with a spray voltage of ±3.8 kV. Capillary temperature was set to 320 °C, sheath gas 40 a.u., AGC target 1x10^6^ a.u. and the lock masses in positive and negative mode were 144.9822 m/z and 100.9856 m/z, respectively.

Fragmentation was performed on pooled samples by isolating ions in a 1.2 m/z window and fragmentation with stepped HCD collision energy of 24.8, 60.0 and 94.8 % for both polarities with 17,500 resolution and AGC target 1x10^5^ a.u. Top 10 ions (intensity threshold 1.3x10^5^) were selected for fragmentation and then added to a dynamic exclusion window for 15 s.

Off-line LC-MS data processing and analysis

Raw mass spectrometry files were converted into .mzXML files in profile mode with the open-source software ProteoWizard (Version 3.0; Chambers et al., 2012). Further data processing and analysis was performed using the Polyomics integrated Metabolomics Pipeline (PiMP) online platform (Gloaguen *et al.*, 2017; date of use: 08 Jul 2020). Peak detection and filtering were set to 3 ppm of the theoretical monoisotopic mass, minimum intensity to 5,000, noise to 0.8, retention window to 0.05 and minimum number of detections to 3. Peak retention time was corrected using the Obiwarp algorithm (Prince & Marcotte, 2006; from xcms package Version 1.48.0).

SUPPLEMENTARY FIGURES


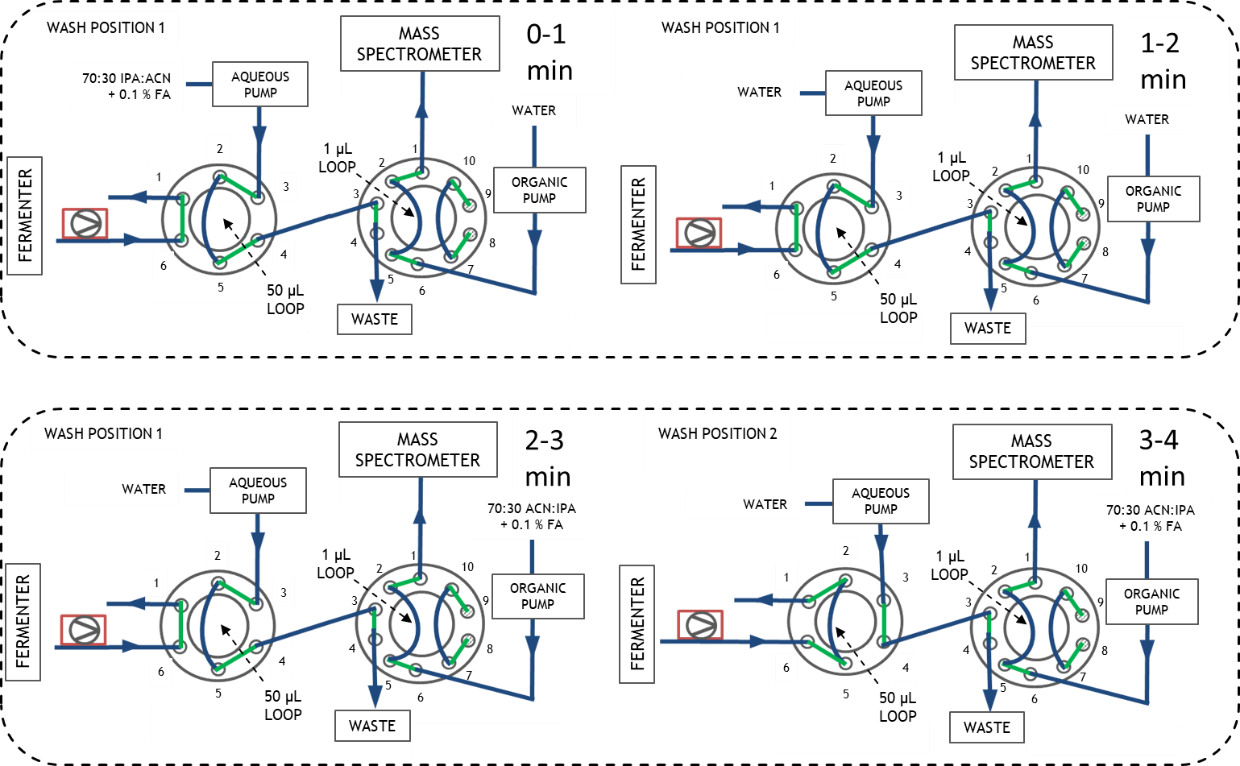


Figure S 1. Diagram showing the positions of the two valves and liquid solutions used during the four-minute washing step between sample injections. The duration of each configuration is indicated on the top right of each diagram. The nomenclature of the pumps (aqueous and organic pump) was based on the solutions used during the injection step.


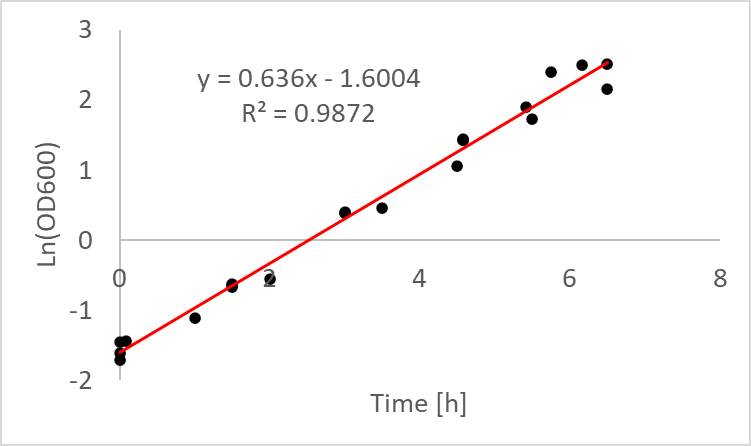


Figure S 2. Natural logarithm of the OD_600_ for four fermentation replicates during the exponential growth phase. The slope of the curve is the exponential growth rate (0.64 h^-1^). The doubling time is calculated as Ln(2)/0.64 = 1.09 h = 65.39 min.


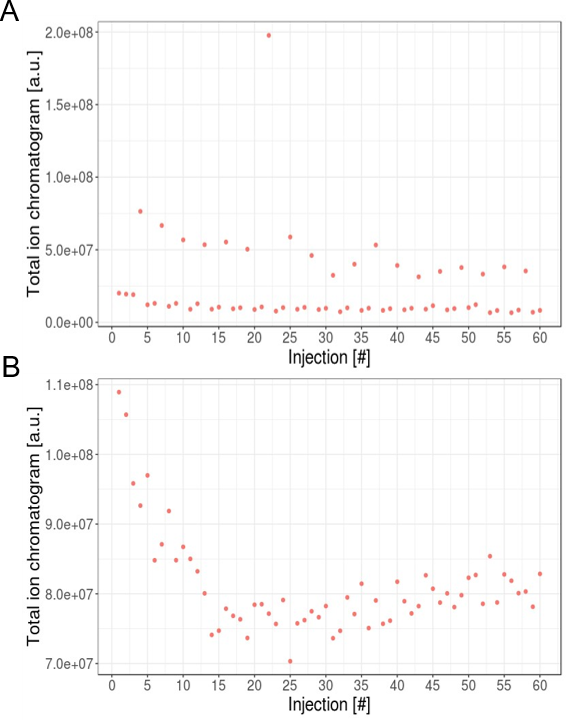


Figure S 3. Total ion chromatogram (TIC) of the first 60 injections from a succinate production fermentation analysed by on-line metabolomics and with a sample:wash ratio of 3:1 (A) or 1:1 (B). Only the first 60 injections are shown for easier visualisation of the x-axis. The TIC increase after every third injection in figure A corresponds to injections immediately after a washing step.

SUPPLEMENTARY TABLES

Table S 1. Conditions tested to develop the on-line metabolomics system. For all experiments, mix A was H_2_O and mix B was 70:30 IPA:ACN + 0.1 % FA. Abbreviations: AC, ammonium carbonate; ACN, acetonitrile; FA, formic acid; IPA, isopropanol; MeOH, methanol.

| Experiment number | Solvent system (mix C) | Six-port valve | Ten-port valve | Frequency of sampling | No. of samples before wash | Comments |
| --- | --- | --- | --- | --- | --- | --- |
| 1 | MeOH | No wash | 3 min B at 200 µL/min  + 7 min C at 200 µL/min | 10 min^-1^ | 12 | System blocked after 5.92 h |
| 2 | MeOH | 2 min B at 200 µL/min  + 7 min A at 200 µL/min | 3 min B at 400 µL/min  + 6 min C at 400 µL/min | 10 min^-1^ | 12 | System blocked after 25.33 h |
| 3 | MeOH | 4 min B at 400 µL/min  + 5 min A at 400 µL/min | 4 min B at 600 µL/min  + 5 min C at 600 µL/min | 10 min^-1^ | 3 | Pressure started to look quite high after 20 h |
| 4 | MeOH | 4 min B at 400 µL/min  + 5 min A at 400 µL/min | 4 min B at 600 µL/min  + 5 min C at 600 µL/min | 10 min^-1^ | 3 | No blockages after 48.60 h but the back-pressure increased after 27.75 h. The TIC was much higher for the samples immediately after a wash step (Figure S3) |
| 5 | MeOH | 4 min B at 400 µL/min  + 5 min A at 400 µL/min | 4 min B at 600 µL/min  + 5 min C at 600 µL/min | 10 min^-1^ | 1 | No blockages or overpressure after  45.83 h |
| 6 | 70:30 ACN: IPA + 0.1 % FA | 1 min B at 400 µL/min  + 3 min A at 400 µL/min | 1 min B at 600 µL/min  + 3 min C at 600 µL/min | 5 min^-1^ | 1 | System blocked after 21.83 h |
| 7 | 70:30 ACN: IPA + 0.1 % FA | 1 min B at 400 µL/min  + 3 min A at 400 µL/min | 2 min A at 600 µL/min  + 2 min C at 600 µL/min | 5 min^-1^ | 1 | System ran for 45.5 h non-stop without blockages |
| 8 | MeOH  + 20 mM AC | 1 min B at 400 µL/min  + 3 min A at 400 µL/min | 2 min A at 600 µL/min  + 2 min C at 600 µL/min | 5 min^-1^ | 1 | System blocked several times after 5.92 h |
| 9 | 70:30 ACN:IPA  + 0.1 % FA | 1 min B at 400 µL/min  + 3 min A at 400 µL/min | 2 min A at 600 µL/min  + 2 min C at 600 µL/min | 5 min^-1^ | 1 | No blockage issues in 47.07 h |
